# Acute splenic sequestration in a pregnant woman with homozygous sickle-cell anemia

**DOI:** 10.1590/S1516-31802013000100023

**Published:** 2013-04-01

**Authors:** Carolina Bastos Maia, Roseli Mieko Yamamoto Nomura, Ana Maria Kondo Igai, Guilherme Hencklain Fonseca, Sandra Menosi Gualandro, Marcelo Zugaib

**Affiliations:** I MD. Postgraduate Student of Obstetrics and Gynecology, Department of Obstetrics and Gynecology, Faculdade de Medicina da Universidade de São Paulo (FMUSP), São Paulo, Brazil.; II MD, PhD. Associate Professor, Department of Obstetrics and Gynecology, Faculdade de Medicina da Universidade de São Paulo (FMUSP), São Paulo, Brazil.; III MD, PhD. Obstetrician and Gynecologist, Department of Obstetrics and Gynecology, Faculdade de Medicina da Universidade de São Paulo (FMUSP), São Paulo, Brazil.; IV MD, PhD. Hematologist, Hospital das Clínicas, Faculdade de Medicina da Universidade de São Paulo (HCFMUSP), São Paulo, Brazil.; V MD, PhD. Full Professor and Chairman, Department of Obstetrics and Gynecology, Faculdade de Medicina da Universidade de São Paulo (FMUSP), São Paulo, Brazil.

**Keywords:** Anemia, sickle cell, Pregnancy, Fetal distress, Fetal monitoring, Spleen, Anemia falciforme, Gravidez, Sofrimento fetal, Monitorização fetal, Baço

## Abstract

**CONTEXT:**

Homozygous (SS) sickle-cell anemia complicated by acute splenic sequestration in adults is a rare event, and it has never been reported during pregnancy.

**CASE REPORT:**

A 25-year-old woman with homozygous (SS) sickle-cell disease was hospitalized at 32 weeks’ of gestation presenting weakness, abdominal pain, fever and hemoglobin of 2.4 g/dl. Abnormal fetal heart rate was detected by means of cardiotocography, and 5 units of packed red cells were transfused. Cesarean was performed at 37 weeks. Both mother and baby were discharged in a good general condition.

**CONCLUSION:**

This case report demonstrates the importance of immediate blood transfusion for treatment of fetal distress in cases of splenic sequestration during pregnancy. This treatment is essential for avoiding maternal and fetal complications.

## RESUMO

**CONTEXTO:**

Anemia falciforme homozigótica (SS) complicada por sequestro esplênico agudo em adultos é evento raro, e nunca foi relatado durante a gravidez.

**RELATO DO CASO:**

Uma mulher de 25 anos, portadora de doença falciforme homozigótica (SS), com 32 semanas de gestação, foi internada apresentando fraqueza, dor abdominal, febre e hemoglobina de 2,4 g/dl. Frequência cardíaca fetal anormal foi detectada pela cardiotocografia e a paciente recebeu 5 unidades de concentrado de hemácias. Cesariana foi realizada com 37 semanas. Mãe e filho receberam alta em bom estado geral.

**CONCLUSÃO:**

Este relato de caso demonstra a importância da transfusão imediata para o tratamento de sofrimento fetal nos casos de sequestro esplênico durante a gestação. Este tratamento é imprescindível para se evitarem complicações maternas e fetais.

## INTRODUCTION

Acute splenic sequestration is a life-threatening event that generally occurs in children up to six years of age. It is uncommon in adults with homozygous (SS) sickle-cell disease.^1^ When a splenic sequestration crisis occurs in an adult with sickle-cell disease, the underlying disease is generally sickle hemoglobin C (SC) disease or sickle-beta thalassemia.^2^ An acute splenic sequestration crisis is defined as a sudden fall in hemoglobin concentration accompanied by reticulocytosis and rapid enlargement of the spleen. It is particularly dangerous during pregnancy, since it can cause an abrupt reduction in fetal oxygenation. Elective splenectomy can be recommended following the first attack of acute splenic sequestration in adults with sickle-cell anemia because of the potentially life-threatening nature of the complication and the high risk of recurrence. However, the procedure is avoided during pregnancy. The low hemoglobin levels during such crises are dangerous to the fetus, since these situations can be complicated by fetal distress and even lead to fetal death. Acute splenic sequestration crises constitute hematological emergencies in pregnant women with sickle-cell disease, and are characterized by worsening anemia and splenomegaly.

We report the case of a patient with homozygous (SS) sickle-cell disease who presented fetal distress and acute splenic sequestration at 32 weeks of gestation. Research on pregnancy complicated by homozygous (SS) sickle-cell disease was approved by the local ethics committee. Acute splenic sequestration crises are unusual during pregnancy complicated by homozygous (SS) sickle-cell disease. Such situations can lead to fetal distress, and the management of the acute fall in hemoglobin levels is discussed.

## CASE REPORT

A 25-year-old woman with homozygous (SS) sickle-cell disease was referred to our tertiary center at 32 weeks of gestation, presenting weakness, fever and worsening anemia. The patient consented to our reporting of the case.

Her obstetric history included a previous pregnancy two years earlier, which had been complicated by mild preeclampsia, and in which cesarean delivery had been performed at term. In the first pregnancy, there were no reports of any complication caused by her sickle-cell disease, such as occurrences of painful crises, splenic sequestration or need for blood transfusion. During the patient’s childhood, her sickle-cell disease had always been relatively mild, with some occasionally painful vaso-occlusive crises and rare admissions to hospital. Hemoglobin electrophoresis performed before the current pregnancy revealed hemoglobin S of 82.2%, hemoglobin F of 15.1% and hemoglobin A2 of 2.7%.

On arrival at our center, she reported experiencing left upper abdominal pain. Her temperature was 38 ºC, heart rate 115 bpm, blood pressure 120/80 mmHg and fetal heart rate 150 bpm. Her spleen was enlarged (palpable 4 cm below the left costal margin), and was tender. The laboratory test results showed hemoglobin level of 2.4 g/dl, hematocrit 7.8%, reticulocyte count 7.43%, white blood cell count 17.8 x 109/l (neutrophils 83%), platelet count 84 x 109/l, alanine aminotransferase 57 U/l, aspartate aminotransferase 94 U/l, total bilirubin 3.63 mg/dl, urea 32 mg/dl, creatinine 1.34 mg/dl and lactic dehydrogenase 1,661 U/l.

Fetal monitoring performed for 60 minutes showed a baseline fetal heart rate of 150 bpm, absence of variability and absence of accelerations. A prolonged deceleration lasting for 4 minutes (80 bpm) was noted at the beginning of the record. The volume of amniotic fluid was normal and the fetal biophysical profile was 6. No fetal Doppler evaluation was performed.

Two packs of red blood cells were immediately transfused. The fetal heart rate pattern progressively returned to normal and the fetal biophysical profile was normal. Twelve hours after the first blood transfusion, the woman’s hemoglobin level was low (4.3 g/dl), and two additional packs of red blood cells were transfused. One day after transfusion, her hemoglobin level was 5.9 g/dl, and one more pack of red blood cells was transfused, thereby increasing her hemoglobin level to 7.4 g/dl.

The patient was kept in hospital. An ultrasound scan showed that fetal growth was normal, and antenatal fetal surveillance examinations were normal. Cesarean delivery was performed at 37 weeks of gestation. The newborn weighed 3,030 grams at birth, and the Apgar scores were 9, 10 and 10 at 1, 5 and 10 minutes. The patient was transfused with two more packs of red blood cells postpartum. Both mother and baby were discharged on the fifth day postpartum, alive and well.

## DISCUSSION

An acute splenic sequestration crisis is a type of anemic crisis, characterized by acute enlargement of the spleen secondary that traps a considerable red cell mass. It leads to acute anemia with or without thrombocytopenia.^1^ Patients whose spleens have not yet developed fibrosis are more susceptible to this disorder. The pathogenesis is not well known. The triggering event may be an acute obstruction of the splenic venous flow with sudden enlargement of the organ, which leads to sequestration of red blood cells and platelets.^2^ The clinical diagnosis of acute splenic sequestration has been defined as a sudden drop of at least 2 g/dl associated with acute splenomegaly and active erythropoiesis. Enlargement of the spleen can lead to increased abdominal girth, abdominal and back pain, circulatory collapse, shock and death.

There are few data on acute splenic sequestration in cases of homozygous (SS) sickle-cell anemia during pregnancy or the puerperium (Table 1). On the other hand, there are few cases of this complication among patients with other hemoglobin diseases. One case of acute splenic sequestration was reported by Noreldeen et al.^3^ A 23-year-old woman with hemoglobin S-beta thalassemia presented a crisis on the sixth day postpartum. In the present case, an elevated level of fetal hemoglobin (15.1%) was observed. The hypothesis that most likely explains the occurrence of acute splenic sequestration in our case is that elevated levels of hemoglobin F may have contributed towards the mildness of the sickle-cell disease and persistence of splenomegaly.

**Table 1.** Search strategies performed on September 15, 2011, and results from each database

Electronic databases	Key words	Results	
		Found	Related
Medline (via PubMed)	(anemia sickle cell) AND (pregnancy) AND (splenic sequestration) OR ( splenic sequestration sickle)	6	None of them demonstrated homozygous (SS) sickle-cell anemia complicated with acute splenic sequestration during pregnancy
Embase	(anemia sickle cell) AND (pregnancy) AND (splenic)	16	None of them demonstrated homozygous (SS) sickle-cell anemia complicated with acute splenic sequestration during pregnancy
	(anemia sickle cell) AND (pregnancy) AND (sequestration)	12	None of them demonstrated homozygous (SS) sickle-cell anemia complicated with acute splenic sequestration during pregnancy
Lilacs	(pregnancy) OR (embarazo) OR (gravidez) AND (anemia sickle cell) OR ( anemia falciforme) OR (anemia de células falciformes) AND (splenic	0	0
	sequestration) OR (sequestro esplênico) OR (secuestro del bazo)		
Cochrane Library (via Bireme)	(anemia sickle cell) AND (pregnancy) AND (splenic)	4	None of them demonstrated homozygous (SS) sickle-cell anemia complicated with acute splenic sequestration during pregnancy
(anemia sickle cell) AND (pregnancy) AND (sequestration)	4	None of them demonstrated homozygous (SS) sickle-cell anemia complicated with acute splenic sequestration during pregnancy

Another two cases have been described during the postpartum period. Black et al.^4^ reported the case of a 24-year-old woman with sickle-cell hemoglobin C disease who died as a consequence of splenic sequestration on the eleventh day postpartum. Solanki et al.^2^ described a 30-year-old woman with sickle-cell ß-thalassemia that presented on the second postpartum day; this patient was discharged in a stable condition after transfusion.

The same authors also described two cases during pregnancy, neither of them with homozygous (SS) sickle-cell anemia. The first case was described by Black et al.,^4^ and involved a 29-year-old pregnant woman with sickle-hemoglobin C disease who presented abdominal pain at 32 weeks of gestation, and acute anemia. A cesarean was performed, with the outcome of fetal death, and the spleen was enlarged. The other case was described by Solanki et al.,^2^ and involved a 24-year-old pregnant woman with sickle-cell hemoglobin C disease complicated by splenic sequestration at 24 weeks of gestation. This case was treated with partial exchange transfusion.

Immediate transfusion of red blood cells is the only effective treatment for acute splenic sequestration, and this should be performed carefully during pregnancy, to avoid adverse maternal and fetal outcomes. It remains unknown whether blood transfusion is the best alternative for restoring fetal wellbeing, but the fetal improvement observed in the present case suggests to us that it made a positive contribution. Severe maternal anemia with hemoglobin levels below 6 g/dl have been associated with abnormal fetal oxygenation,^5^ thereby resulting in abnormal fetal heart rate patterns, fetal cerebral vasodilatation and reduced amniotic volume.^4^ In the present case, severe acute maternal anemia caused fetal hypoxemia, and fetal heart rate abnormalities that were detected by means of cardiotocography. It is likely that, if the maternal anemia had persisted, fetal cerebral injury could have taken place, or fetal death. The blood transfusion corrected the maternal anemia and improved the fetal condition. Elective splenectomy should be recommended following the first acute splenic sequestration crisis in adults with sickle-cell disease,^6^ since the latter is a life-threatening event and is recurrent in 50% of survivors.

## CONCLUSION

We reported on a case of acute splenic sequestration during pregnancy, at 32 weeks of gestation, in a patient with homozygous (SS) sickle-cell disease. Acute maternal anemia was the leading cause of transient fetal distress.

Immediate transfusion of red blood cells is the treatment for fetal distress in cases of acute splenic sequestration during pregnancy. It should be performed promptly so that adverse maternal and fetal outcomes can be avoided. Obstetricians need to work closely with hematologists in order to prevent maternal or fetal adverse outcomes in cases of acute splenic sequestration crisis during pregnancy.
